# Bisphosphonate Combination Therapy in the Management of Postchemotherapy Avascular Necrosis of the Femoral Head in Adolescents and Young Adults: A Retrospective Study From India

**DOI:** 10.1200/JGO.17.00083

**Published:** 2018-01-30

**Authors:** Sanjay Agarwala, Shripad D. Banavali, Mayank Vijayvargiya

**Affiliations:** **Sanjay Agarwala** and **Mayank Vijayvargiya**, P.D. Hinduja Hospital and Medical Research Centre; and **Shripad D. Banavali**, Tata Memorial Centre, Mumbai, India.

## Abstract

**Purpose:**

With improved survival after chemotherapy for acute lymphoblastic leukemia (ALL), it is imperative to maintain good quality of life as part of the management of post-therapy adverse effects. Avascular necrosis of the femoral head (AVNFH) is one such adverse effect. A need exists for a therapy that ameliorates discomfort, provides a productive life, is cost effective, and is joint preservative. We conducted the current study to evaluate the response to bisphosphonate in the nonsurgical management of AVNFH in adolescents and young adults (AYA) who receive treatment for ALL.

**Materials and Methods:**

This is a retrospective study of 20 AYA patients—34 affected hips—who received zolendronic acid 5 mg intravenously each year along with oral alendronate 70 mg weekly for 3 years. Clinical evaluation was performed by using the Visual Analog Scale and the Harris Hip Score. Radiographs were used to classify the Ficat-Arlet stage, monitor radiologic collapse, and evaluate the rate of progression.

**Results:**

Pain relief with a drop in the Visual Analog Scale score was observed at a mean duration of 5.2 weeks (range, 3 weeks to 11 weeks) after the start of therapy. Radiologic progression by one grade was observed in 12 hips (35.3%), and only one hip (2.94%) showed progression by two grades. At a mean follow-up of 50.3 months, 31 affected hips (91.1%) had a satisfactory clinical outcome and had not required any surgical intervention. The proportion of hips that required total hip arthroplasty were 0%, 5%, and 22.2% in Ficat-Arlet stage I, II, and III, respectively.

**Conclusion:**

The combination of intravenous zolendronic acid and oral alendronate provides a pragmatic solution for the management of AVNFH after therapy for ALL in AYA patients. This therapy is safe, effective, and well tolerated.

## INTRODUCTION

Because the treatment of acute lymphoblastic leukemia (ALL) now yields 5-year overall survival rates > 90%, there is a need for strategies for assessing the burden of toxicities in the overall evaluation of antileukemic therapy programs.^1^ Avascular necrosis (AVN)/osteonecrosis (ON) is one of the most common therapy-related and debilitating adverse effects of antileukemic treatment and can adversely affect long-term quality of life.^2^

The incidence of AVN in children and adolescents who undergo chemotherapy for ALL varies from 2.3% to 7.7%.^3-6^ Median age at diagnosis of AVN was 13.5 years in one study.^7^ Median time from diagnosis to the development of AVN after chemotherapy is 1.14 years (range, 0.25 years to 2.12 years).^4,5^ Whereas the etiology of AVN is multifactorial, independent risk factors that have been found to be associated with the development of AVN are female sex,^7^ high body mass index,^8^ PAI-1 (SERPINE1) genetic variation,^9^ lower albumin,^10^ high cholesterol and lipid levels,^10,11^ and genomic variation.^10,12^

The objective of treating avascular necrosis of the femoral head (AVNFH), especially in adolescents and young adults (AYAs), is to prevent disease progression and the collapse of the femoral head, obtain pain relief, and restore joint movement. Unfortunately, no standard of care exists for the treatment of ALL-associated AVNFH. Whereas considerable practice variation exists, surgical intervention seems to be relatively safe and is still the treatment of choice.^3,13^ The only surgical modality that has shown good results is the total hip arthroplasty (THA); however, THA performed at a young age will necessitate at least one revision arthroplasty in the future, as the average life expectancy of the patient exceeds THA survivorship.^14,15^

Research on the medical management of AVN with bisphosphonate (BP) has shown promising results, whereas other therapies have failed.^4,5,16-20^ BP improved pain scores, analgesic requirement, and musculoskeletal function in patients with ON that occurred as a complication of childhood ALL therapy, but an objective radiologic benefit could not be demonstrated.^17^ Despite treatment with single-agent zolendronic acid (ZA), most patients with AVNFH experienced progressive joint destruction that required arthroplasty.^4^ Novel treatment strategies are therefore needed to prevent this debilitating complication in survivors of childhood cancer.^4^

The challenge with BPs is to develop the ideal regimen that has a faster onset of action, good tolerability, and provides a better clinico-radiologic outcome. Agarwala et al,^21^ in a prospective trial of 60 patients with AVN (100 hips), reported that the use of oral alendronate retards progression, prevents collapse, improves clinical outcomes, and potentially avoids arthroplasty. Studies have shown that the suppression of bone resorption occurs approximately within 3 months of the initiation of alendronate, but is more rapid after intravenous ZA administration.^22^ In addition, a biomarker evaluation by Saag et al^23^ demonstrated detectable levels of urine N-terminal telopeptide at 1 week after ZA administration, but with alendronate, the same was detectable at 12 weeks. This indicates that ZA, with its higher bioavailability and faster onset of action, complements alendronate therapy.

On the basis of this background and our clinical experience, we believe that the combination of oral alendronate and intravenous ZA could successfully treat AVN in AYA patients, ameliorate discomfort, and provide a productive life. Furthermore, this treatment is cost effective and joint preservative. We conducted this study to evaluate the clinicoradiologic outcome of the management of AVNFH with ZA and alendronate in adolescent patients with ALL.

## MATERIALS AND METHODS

This retrospective study was conducted at a tertiary care hospital in Mumbai, India. Institutional review board approval was obtained. Data on patients with ALL age 13 to 25 years—adolescents and young adults—who were treated for AVNFH between January 2009 and December 2013 were recorded. Patients with hip arthritis (Ficat-Arlet stage IV),^24^ renal or hepatic dysfunction, and a known allergy or intolerance to ZA or alendronate were excluded. A total of 28 patients (46 hips) with AVNFH were treated at our center during the study period, of which we exluded four patients (six hips) with stage IV AVNFH, two patients (three hips) who were lost to follow-up, and two patients (three hips) with intolerance to BP therapy; 20 patients (three hips) are included in the study after applying the exclusion criteria. Mean age at presentation was 18.4 years (range, 14 to 24 years). There were 17 and three male and female patients, respectively. Mean follow-up was 50.35 months (range, 37 to 85 months).

### Assessment

Only symptomatic patients were evaluated for AVN using radiographs and magnetic resonance imaging (MRI). Demographic data, the time interval between the diagnosis of ALL and the onset of AVN, pain Visual Analog Scale (VAS), and the Harris Hip Score^25^ (HHS) were recorded. All patients were observed at 3 weeks and 6 weeks and every 3 months thereafter in the first year, then annually for a minimum of 3 years. Radiologic assessment was performed with plain radiographs of both hips in anteroposterior and lateral views at every visit. Ficat-Arlet classification^24^ was used to classify AVNFH by the same radiologist at every visit. MRI was performed at the time of presentation to diagnose suspected Ficat-Arlet stage I AVN and was not repeated at every visit for financial reasons.

Clinical failure was noted to occur when pain and disability warranted surgical intervention. Radiologic failure was assigned as a radiologic progression of osteonecrosis by one to two grades/stages per Ficat-Arlet staging. Radiologic collapse was noted to occur when progression from Ficat-Arlet stage I or II to stage III was observed.

### Medical Management

Patients received intravenous ZA (5 mg) at the first visit and on a yearly basis thereafter, and oral alendronate 70 mg weekly was administered for 3 years. All patients received oral daily supplements of calcium 500 mg and vitamin D 400 IU. Analgesics were administered as and when required. Partial weight bearing using axillary or elbow crutches was advised for the first 3 months after the initiation of BP therapy, and weight bearing was allowed as tolerated thereafter.

### Statistical Analysis

Statistical analysis was performed using SPSS for Windows version 20.0 (SPSS, Chicago, IL). We used the Wilcoxon signed-rank test to determine the level of significance between groups after confirming the normal distribution of results using the Shapiro-Wilk test. *P* values < .05 were considered significant.

## RESULTS

The mean duration of hip pain before diagnosis was 8.06 months (range, 2 to 21 months; Table 1). All patients received the MCP 841 chemotherapy protocol for ALL.^26^ The total duration of chemotherapy was 2 years. The cumulative dose of prednisolone was 4,000 mg/m^2^. Prednisolone was stopped once AVN was diagnosed. The mean duration of chemotherapy before AVN diagnosis was 12.53 months (range, 6 to 28 months).

**Table 1:**
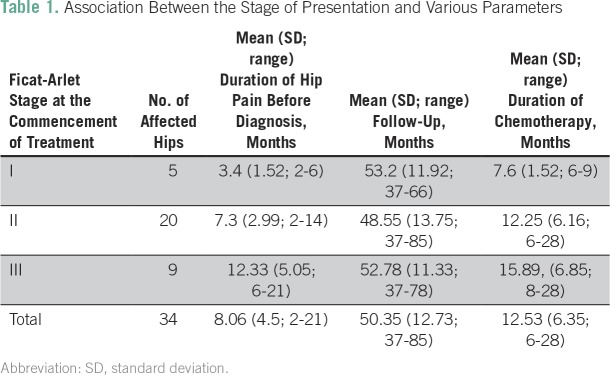
Association Between the Stage of Presentation and Various Parameters

### Clinical Outcome

At a mean follow-up of 50.35 months (range, 37 to 85 months), 31 hips (91.1%) had a satisfactory clinical outcome and did not require any surgery. Clinical failure that required THA occurred in one (5%) of 20 in stage II hips and two (22.2%) of nine in stage III hips. There was no clinico-radiologic failure that required THA in stage I hips.

**Fig 1:**
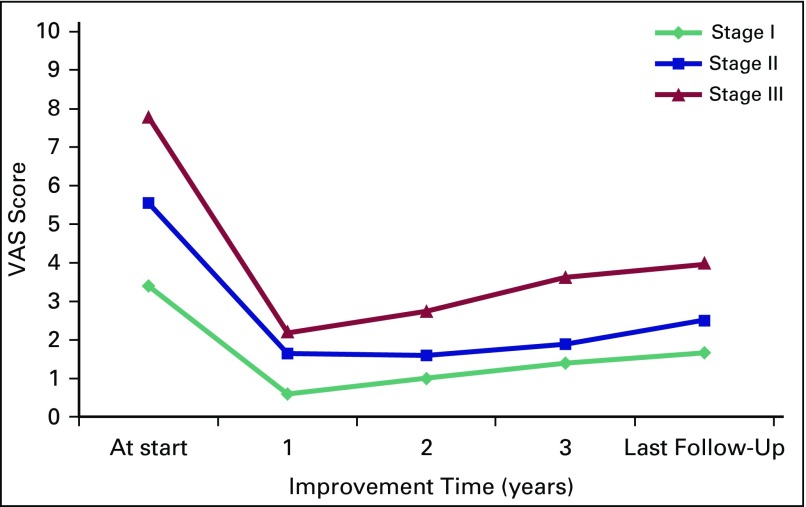
Line graph demonstrating the improvement in stage-wise Visual Analog Scale (VAS) pain scores compared with baseline scores at subsequent years.

**Fig 2:**
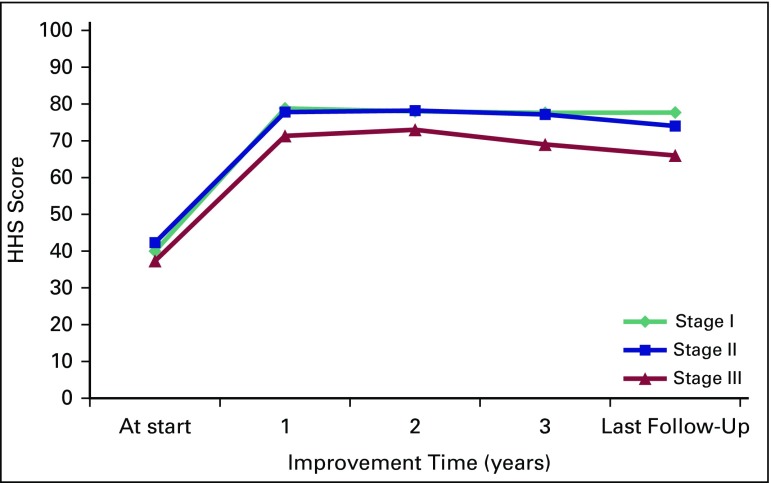
Stage-wise mean Harris Hip Score (HHS) in the form of graph to show the improvement in yearly scores recorded.

Clinical outcome with regard to VAS score and HHS is presented in Figures 1 and 2, respectively. Improvement in mean VAS pain score occurred from 5.82 at the start of therapy to a score of 2.72 in a mean duration of 5.2 weeks (range, 3 to 11 weeks). The lowest value of mean VAS score was noted at 1 year, with a slight increase at subsequent follow-ups, but it remained significantly lower compared with VAS score at presentation. Corresponding to VAS score, the mean analgesic requirement also decreased significantly. A similar pattern was observed in mean HHS, which peaked at 1 year, then gradually declined but remained significantly higher than HHS at presentation. Clinical outcome significantly improved after the commencement of therapy in all stages (*P* < .001; Table 2).

**Table 2:**
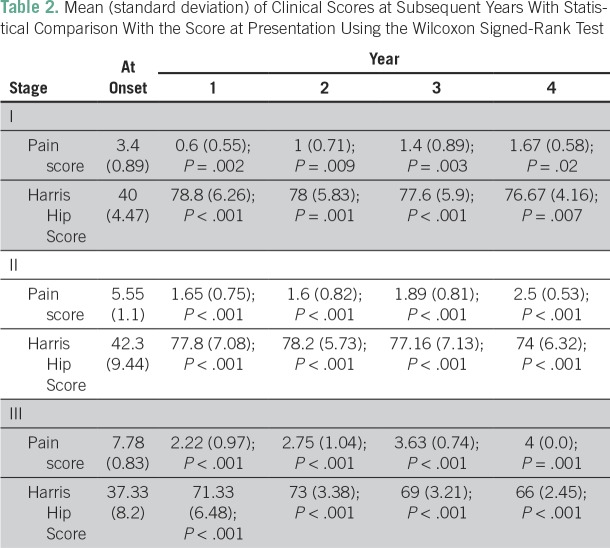
Mean (standard deviation) of Clinical Scores at Subsequent Years With Statistical Comparison With the Score at Presentation Using the Wilcoxon Signed-Rank Test

### Radiologic Progression

**Fig 3:**
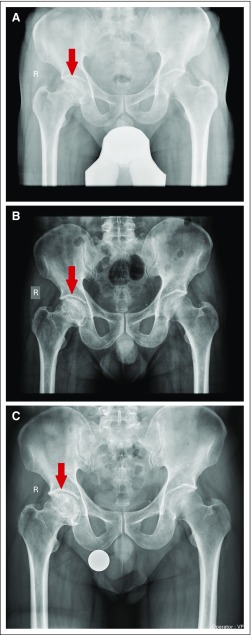
(A) Radiograph of a 22-year-old patient at the start of therapy showing Ficat-Arlet stage II avascular necrosis of right femoral head. (B) Follow-up radiograph at 1 year showing no progression. (C) Pelvis radiograph at 7 years showing no progression/collapse of the femoral head and good radiologic outcome.

**Fig 4:**
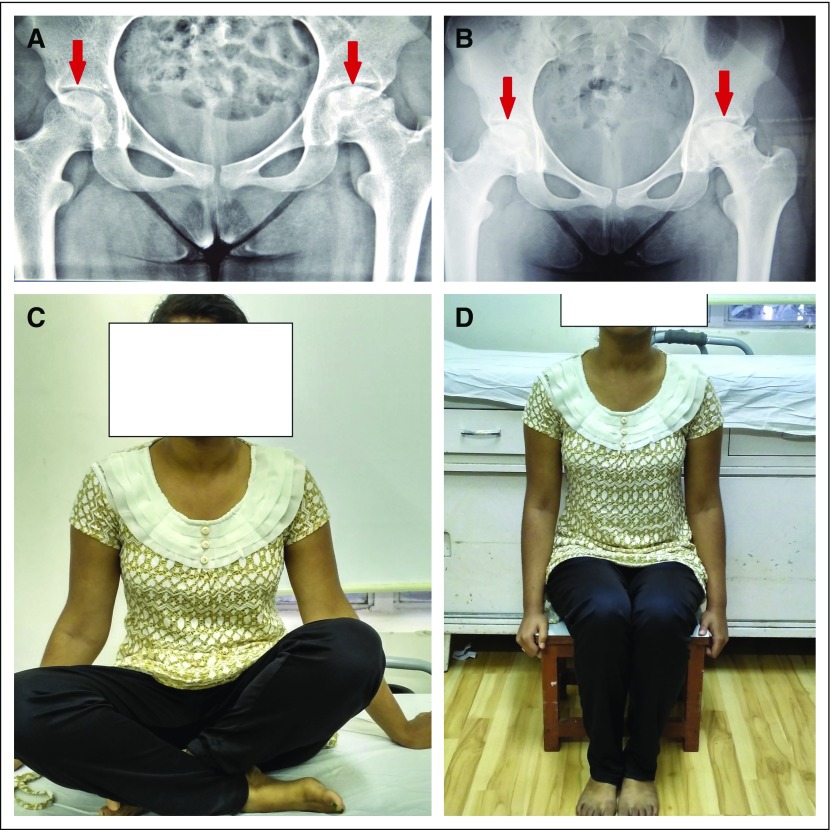
(A) Radiograph of a 19-year-old patient showing Ficat-Arlet stage II avascular necrosis (AVN) right femoral head and stage III AVN left femoral head at the commencement of therapy. (B) Radiograph at year 4 of follow-up demonstrating no radiologic progression of the AVN stage on both the sides. (C and D) Patient is able to squat and sit on a low stool, showing no limitation of daily activities.

Radiologic progression was observed in 13 (38.23%) of 34 affected hips at a mean follow-up of 50.35 months. Progression by two grades was seen in one hip (2.94%), and progression by one grade was seen in 12 hips (Table 3). Two (40%) of five hips in stage I, eight (40%) of 20 in stage II, and three (33.3%) of nine in stage III demonstrated progression. Radiologic collapse—that is, progression from stage I or II to stage III—was seen in nine (26.47%) of 34 hips. All cases that demonstrated collapse belonged to stage II, with the exception of one case that belonged to stage I. Mean time to collapse was 25.7 months (range, 10 to 42 months). Figures 3 and 4 show the improvement in outcomes after BP therapy in different stages.

**Table 3:**
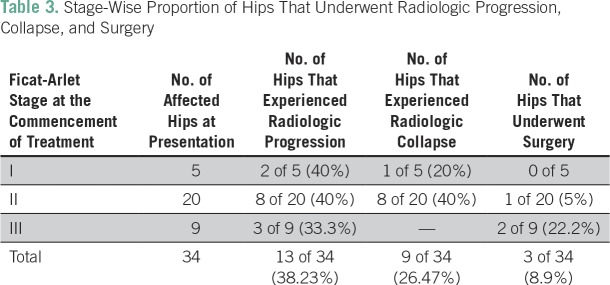
Stage-Wise Proportion of Hips That Underwent Radiologic Progression, Collapse, and Surgery

### Adverse Effects

Two patients (10%) experienced flu-like symptoms after receiving ZA, which lasted for a maximum of 2 days. Of the 60 ZA infusions administered (each patient received three infusions of ZA), flu-like symptoms were noticed on only two occasions, and for both patients, it was the first infusion. Subsequent infusions in both patients did not evoke any such reactions. Mild dyspeptic symptoms were reported in three patients (15%) who received oral alendronate. It did not require the cessation of therapy and resolved within 1 month of the start of therapy. We have already excluded those patients (two) who were intolerant to BP therapy and required the cessation of therapy.

## DISCUSSION

Cure rates for ALL have improved, but as the therapy has intensified, the burden of ON has increased.^3^ Osteonecrosis is one of the 14 acute toxic effects that are serious, but too rare to be addressed comprehensively within any single group.^27^

One of the great challenges is to identify interventions that will reduce the frequency and severity of long-term toxicities without adversely affecting cure rates. Use of alternate-week dosing of dexamethasone—to reduce the risk of ON—is an example of such a successful strategy.^28^ A novel approach to reduce ON-associated morbidity might be systematic early screening for ON by serial MRI, especially in children older than age 10 years who received dexamethasone^2,29^; however, this would not be practical in most low- and middle-income countries.

Current treatment of AVNFH includes analgesia, protected weight bearing, medical management, and such surgical procedures as THA. Various medical therapies, such as hyperbaric oxygen therapy, prostaglandin infusions, low-molecular-weight heparin, and statins, have been evaluated but have demonstrated inconsistent results.^19,20,30-32^ The successful use of BPs for the treatment of AVNFH in adults was first reported by Agarwala et al.^33^ On a cellular level, BPs retard the osteoclastic resorption of necrotic bone during repair, thereby helping in maintaining femoral head sphericity and allowing for the revascularization and prevention of the collapse of the femoral head. Several studies have subsequently reported good outcomes with BPs.^21,34-38^ To our knowledge, there are five published studies on BP use for AVN in pediatric patients with ALL.^4,5,16-18^

Padhye et al^4^ evaluated ZA in the treatment of ON in 18 patients with ALL. At final follow-up, two patients were pain free, three underwent arthroplasty, and 13 were still symptomatic. Leblicq et al^5^ reported pain improvement in 77% of patients with AVN and functional improvement in 59% patients at 1 to 4 months after pamidronate therapy. In another study, the authors concluded that pamidronate had a palliative effect with pain relief but did not prevent late bony collapse and joint destruction.^17,18^ These studies demonstrate that, on a long-term basis, intravenous BPs are not effective in preventing radiologic progression and collapse of the femoral head, but helped to cause a significant improvement in pain and clinical scores acutely.^4,5,17,18^

Greggio et al^16^ reported two cases of AVNFH in ALL that were treated with short-term alendronate therapy—10 mg a day for 12 months—and that showed an improvement in pain and joint mobility until 7 years of follow-up. Although good results were observed, the sample size is too small to draw any conclusions. Compared with the above studies, in our study, with adequate follow-up (mean, 50.35 months), a satisfactory result—that is, no requirement of surgical intervention—was seen in 31 (91.1%) of 34 hips. Proportions of hips that required THR were 0%, 5%, and 22.2% in Ficat-Arlet stage I, II, and III, respectively. Intravenous ZA was preferred over intravenous pamidronate in our study, because, first, it required a yearly single dose and shorter infusion time, and second, because studies have reported inconsistent results with pamidronate.^5,17,18^ Third, in vivo and in vitro studies have reported ZA to be 500 times more potent than pamidronate in the prevention of skeletal-related events.^39^

Yuan et al^40^ conducted a recent meta-analysis of five randomized controlled trials that evaluated the role of BP in AVNFH in a total of 329 participants with 920.9 patient-years of follow-up. They reported that, with the exception of one study, all have demonstrated no clinico-radiologic improvement. Winkel et al,^41^ in a review article, reported that studies on the treatment of ON provided low-quality evidence, and thus concluded that preventing the development of ON is recommended. Kaste et al,^42^ in a review article, also demonstrated that longitudinal outcome studies are limited, and a standardized treatment protocol for the management of AVN in patients with ALL is not yet defined. Vora et al^12^ concluded that few studies have shown a promising role for BPs, but their efficacy in preventing progression is still uncertain.

Heneghan et al^3^ conducted a multicenter study to understand the natural history of AVN in patients with ALL. In a cohort of 10,729 patients with ALL, 242 (2.33%) developed AVN within 5 years of diagnosis. Of 242 patients, 55 patients (22.7%) underwent surgical intervention; therefore, the authors concluded that many patients require surgery for AVN.^3^ Winkel et al reported that, if left untreated, after a mean follow-up of 5 years, 60% of patients had persistent symptoms.^7^ In only 25% of patients, lesions improved radiologically, and 18% of patients underwent surgery.^7^ In our study, the proportions of hips that required THA were 0%, 5%, and 22.2% for stage I, II, and III disease, respectively, compared with 65%, 69%, and 87%, respectively, as reported by Mont and Hungerford, in untreated adult hips.^43^

Many studies have demonstrated the effectiveness of alendronate in improving clinicoradiologic outcome, but improvement in pain and function is observed late.^35-38^ Agarwala et al^38^ demonstrated that, at a 10-year follow-up, 46 (87%) of 53 adult hips did not require arthroplasty, thus concluding that alendronate administered for 3 years maintained its beneficial effect for 10 years. In the current study, improvement in pain and a drop in analgesic requirement was observed as early as 3 weeks, with the mean duration being 5.2 weeks (range, 3 to 11 weeks). Previous studies on oral alendronate only therapy had reported an improvement in pain the earliest—at 12 weeks.^38^ These studies indicate that the combination of oral alendronate with ZA provides a comparatively earlier onset of pain relief.

Some authors have attributed the good results observed in the management of traumatic osteonecrosis of the femoral head in adolescents to the early initiation of therapy and a higher dose of BPs.^44^ Similarly, a dose-related increase in bone mineral density has been observed with alendronate in the management of osteoporosis.^45^ Studies have shown that BP distribution is less in necrosed bone compared with the revascularizing femoral head. Thus, to achieve a sufficient concentration in necrosed bone, continued administration of the drug is required until revascularization has occurred.^46^

A higher dose or longer duration of BP therapy must be balanced against adverse effects. Generally, severe adverse effects that are associated with BP therapy are uncommon in the pediatric population.^44,47,48^ Osteonecrosis of the jaw (ONJ) is a reported complication seen with BP use in adults, especially in patients who undergo dental/surgical procedures of the jaw while receiving BP.^49^ Felsenberg et al^50^ reported no case of ONJ in a clinical trial of 17,000 patients who received oral BP. To date, there have been no cases reported in children or adolescents.^40^ Recommendations to reduce ONJ risk include the completion of oral surgery before the initiation of BP therapy, use of antibiotics before and/or after the oral procedure, appropriate wound closure after tooth extraction, and good oral hygiene.^51^

Animal studies have shown growth disturbances with the use of potent BPs,^52^ but the occurrence of such an effect is lacking in human studies.^53,54^ In our study, we did not observe any major adverse effect that necessitated the withdrawal or stoppage of treatment. No case of uveitis, renal compromise, or ONJ has been reported. Similarly, no adverse effects were reported by Ramachandran et al^44^ in the management of traumatic osteonecrosis in adolescents to whom they administered ZA at a dose of 0.025 to 0.05 mg/kg. The average number of infusions administered was 8.1 (range, seven to nine). A high dose of ZA—4 mg every 3 to 4 weeks—is routinely used in the prevention of skeletal-related events in the management of cancer without major adverse effects.^55^ Thus, the amount of ZA—5 mg per year for 3 years—used in our study is far less than the amount used for the prevention of skeletal-related events and was found to be safe.

The combination of intravenous pamidronate—120 mg divided in 3 to 4 doses administered over 2 weeks—and oral alendronate—70 mg administered for 4 to 6 months—has already been used by Kraenzlin et al^56^ for knee osteonecrosis treatment. This was based on the principle that the prevention of necrotic bone resorption during revascularization is dose dependent.^57^ The addition of intravenous BP resulted in rapid pain relief, with VAS decreasing from 8.2 ± 1.2 at the start of therapy to 5.02 ± 0.6 after 4 to 6 weeks (*P* < .001). Therapy was well tolerated, with all patients completing therapy.

One major limitation of this study is that it lacks a control group that received no BPs. Although series with treatment alone have demonstrated improvement in the outcome of AVN with the use of BP, many controlled studies have failed to show any improvement in outcome.

In conclusion, the combination of yearly intravenous ZA and oral alendronate provides a pragmatic solution for the management of AVN for postchemotherapy ALL in AYAs. It not only provides early pain relief, but also prevents long-term radiologic progression, thereby obviating the need for surgery. Of our patients at various stages of AVN, 91.1% demonstrated good clinical improvement. This combination is well tolerated. If we keep in mind the possibility of AVNFH in AYA and take precautions to prevent it or make efforts to diagnose it earlier, by using the treatment mentioned herein, we may totally avoid the need for THA in these young patients. Thus, we present a new paradigm in the management of a condition that lacks standard management guidelines.
